# Geometric Data Perturbation-Based Personal Health Record Transactions in Cloud Computing

**DOI:** 10.1155/2015/927867

**Published:** 2015-02-12

**Authors:** S. Balasubramaniam, V. Kavitha

**Affiliations:** Department of Computer Science Engineering, University College of Engineering, Anna University, Kanchipuram, Chennai, Tamil Nadu 631501, India

## Abstract

Cloud computing is a new delivery model for information technology services and it typically involves the provision of dynamically scalable and often virtualized resources over the Internet. However, cloud computing raises concerns on how cloud service providers, user organizations, and governments should handle such information and interactions. Personal health records represent an emerging patient-centric model for health information exchange, and they are outsourced for storage by third parties, such as cloud providers. With these records, it is necessary for each patient to encrypt their own personal health data before uploading them to cloud servers. Current techniques for encryption primarily rely on conventional cryptographic approaches. However, key management issues remain largely unsolved with these cryptographic-based encryption techniques. We propose that personal health record transactions be managed using geometric data perturbation in cloud computing. In our proposed scheme, the personal health record database is perturbed using geometric data perturbation and outsourced to the Amazon EC2 cloud.

## 1. Introduction

Cloud computing is an infrastructural paradigm shift that is sweeping through the world of enterprise information technology (IT). Cloud computing is a collection of utilities built using Internet technologies that offer on-demand services over the Web. Users no longer need to have expertise in or control over the technology infrastructure in the cloud. Currently, cloud computing has as many definitions as there are squares on a chessboard [1]. Buyya's scientific definition of cloud computing states that the cloud is a market-oriented distributed computing system, which consists of a collection of interconnected, virtualized computers that are dynamically provisioned and presented as one or more unified computing resources based on service-level agreements established through negotiations between the service provider and consumers [2]. The National Institute of Standards and Technology in the United States has proposed three cloud computing service models: software as a service, platform as a service, and infrastructure as a service [3]. New service models may be derived from these three basic cloud service models and are referred to as specific cloud service models. Cloud computing introduces the concept of “everything as a service,” in which the different components of a system can be delivered, measured, and consequently priced as a service: for example, IT infrastructure, platform development, and databases [2]. Despite all the advantages offered by cloud computing, several challenges hinder the migration of customer software and data to the cloud. Prime among them are data security and privacy issues arising from the outsourcing and processing of sensitive data on remote machines, which are not owned or even managed by the customers themselves [4].

A personal health record (PHR) service allows patients to create, manage, and control their personal health data in one place through the Web. The service allows efficient storage, retrieval, and sharing of medical information; in particular, each patient is guaranteed full control over their medical records, and patients can share their health data with a wide range of users, including healthcare providers, family members, and friends. Owing to the high cost of establishing and maintaining specialized data centers, many PHR services are outsourced to or provided by third-party service providers, for example, Microsoft Health Vault [5]. However, by storing PHRs in the cloud, patients lose physical control over their personal health data, which makes it necessary for each patient to encrypt their PHR data before uploading to cloud servers [6]. Several encryption techniques have been discussed earlier [7]. These techniques are based on conventional cryptographic primitives, such as symmetric key cryptography [8–10], public key cryptography [9, 11], attribute-based encryption (ABE) [12, 13], and cipher text policy ABE (CP-ABE) [14]. However, key management issues remain largely unsolved with these cryptography-based encryption techniques. The risks and crises for health care providers have been analyzed based on the organizational and human aspects, clinical, IT-related, and utilities-related risks [15].

Data perturbation is one of the major techniques for preserving privacy. It is very useful for data owners who wish to publish data while preserving privacy-sensitive information [16]. Before the data owners publish the data, they can change the data in certain ways to distinguish sensitive information while preserving the original information. Several perturbation techniques have recently been proposed for mining purposes; some of them are the randomization approach [17], *K*-anonymization [18], the random rotation perturbation approach [19, 20], and condensation approach [21]. An ideal data perturbation algorithm aims at minimizing privacy loss and information loss [16]. However, these two factors are not well balanced with most existing perturbation techniques. Compared with other approaches to privacy-preserving data mining, geometric data perturbation significantly reduces the complexity in balancing data utility and guaranteeing data privacy: both data utility and privacy guarantee are well preserved [22]. Geometric data perturbation (GDP) consists of a sequence of random geometric transformation, including multiplicative transformation (*R*), translational transformation (Ψ), and distance perturbation (noise additive component):

(1) $$\begin{array}{c} G\left(X\right)=RX+\Psi +\Delta . \end{array}$$

The component *R* can be a rotation matrix [19] or random projection matrix [20]. The component Ψ can be translation transformation; the third component can be a random matrix Δ, which is referred to as a noise additive component [22]. In this paper, we propose GDP-based PHR transactions in cloud computing. In our proposed scheme, the PHR database is perturbed using GDP and outsourced to the Amazon EC2 cloud. During the outsourcing, the cloud receives no details of the original private PHR data.

Our contributions can be summarized as follows.

- For the first time, we have enabled PHR transactions to be made using GDP, and the transactions can be efficiently performed by securely outsourcing the PHR database.
- We conducted experiments on local server and real cloud Amazon EC2 platform to validate our proposed scheme.

The rest of the paper is organized as follows. In Section 2, we discuss related work, and this is followed by the detailed description of our proposed scheme in Section 3.  Section 4 presents implementation results and performance analysis discussed in Section 5; the paper is concluded in Section 6.

## 2. Related Work

Outsourced data may be secured on semitrusted servers based on the symmetric key derivation method, but this approach is poorly scalable [8]. Files in a PHR are organized by hierarchical categories, which make key distribution more efficient, but user revocation is not supported [9]. A binary key tree is constructed over the block keys to reduce the number of keys given to each user [10]. In symmetric key-based encryption, the key management overhead is high if there are large numbers of users and owners. With hierarchical identity-based encryption, each category label is regarded as an identity [9], but this also has a potentially high key management overhead. Both symmetric key-based and public key-based encryption techniques suffer from low scalability for large databases.

To avoid these problems, one-to-many mapping encryption methods, such as attribute-based encryption, may be used [12]. An example of these is CP-ABE for sharing PHR data [14], though certain key management issues remain unsolved. Using a framework of access control to achieve patient-centric privacy for PHRs in cloud computing based on ABE reduces the complexity of key management [6]. ABE techniques may be employed to encrypt each patient's PHR file and provide secure sharing of PHRs in cloud computing; they support on-demand user or attribute revocation and multiauthority ABE [5]. Third-party auditing (TPA) may be employed to verify the cloud server which is used to store and process the PHRs, and homomorphic encryption with data auditing can verify the trustworthiness of the TPA [23]. Controlled secure aggregation protocol using lattice based homomorphic encryption has been designed for enhancing both privacy and accuracy when patients outsource their clinical data for sharing [24]. Advanced encryption standard (AES) is used widely nowadays for security of cloud. When migration of data to the chosen cloud service provider (CSP) happens and in future whenever an application uploads any data on cloud, the data will first be encrypted using AES algorithm and then sent to the CSP [25, 26].

The random rotation-based multidimensional perturbation approach for privacy-preserving data classification in data mining perturbs multiple columns in one transformation [19]. The present study uses random projection matrices for privacy-preserving data mining [20]. An individually adaptable perturbation model enables individuals to choose their own privacy levels [27]. The geometric perturbation approach has been used for preserving the privacy of multiparty collaborative data mining [28]. This study proposed a method based on a geometric data perturbation privacy-preserving data classification: it includes rotation perturbation, translation perturbation, and distance perturbation. Both data utility and data privacy guarantees are well preserved compared with other perturbation-based approaches [22]. This paper explains random space data perturbation method to provide range query and *k*-nearest neighbor query services for protected data in the cloud. Random space data perturbation combines order-preserving encryption, dimensionality expansion, random noise injection, and random projection [29].

## 3. Proposed Work

**Figure 1:**
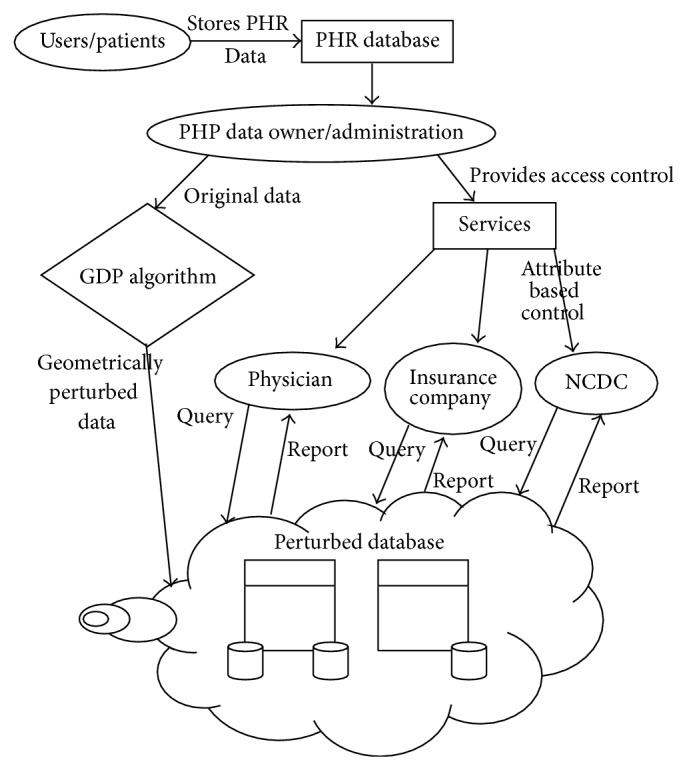
System architecture.

In our proposed work, we perform PHR transactions using GDP in cloud computing. Our work consists of three stages: GDP algorithm, access control policy, and data retrieval/query processing. Our system architecture appears in Figure 1.

### 3.1. PHR Database

**Figure 2:**
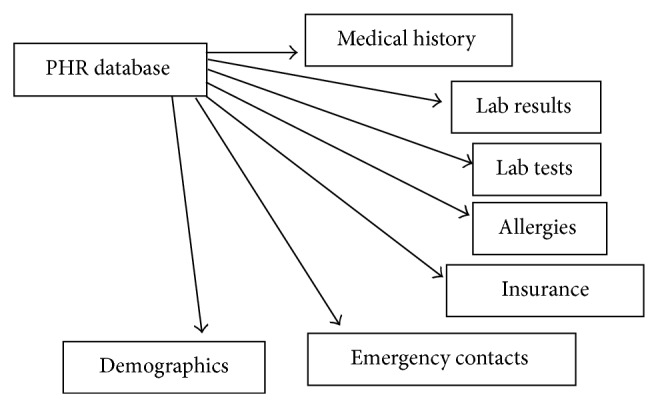
PHR database.

**Figure 3:**
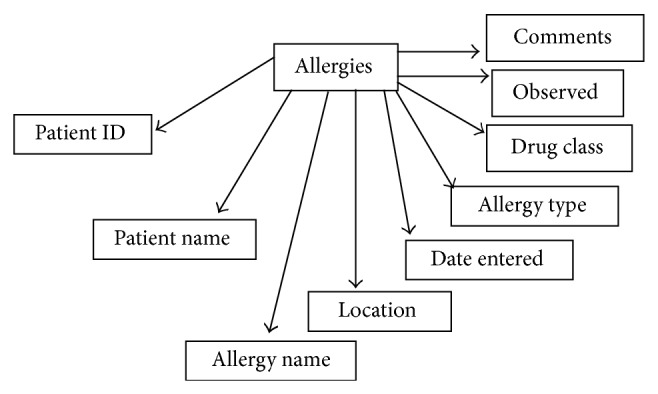
Allergies table.

In our application, the PHR database contains various types of information about the patient. The following information about the patient is available in our PHR database, as shown in Figure 2: demographics, emergency contacts, insurance details, allergies, lab tests, lab results, and medical history. For each area of information, we maintain a separate table, and each table consists of different attributes (columns), as shown in Figure 3. For example, the allergies table consists of patient ID, patient name, allergy name, location, date entered, allergy type, drug class, observed details, and comments.

**Figure 4:**
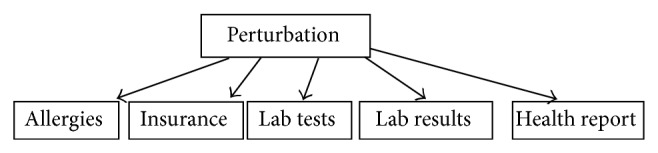
Perturbation table.

Similarly, all the tables consist of different patient attributes. With the GDP algorithm, we have the option of choosing any one of the five tables—allergies, health report, insurance details, lab tests, and lab test results—as shown in Figure 4. It is also possible to choose any number of columns for a particular table for the perturbation process. The various columns available in the allergies table have already been indicated.

**Figure 5:**
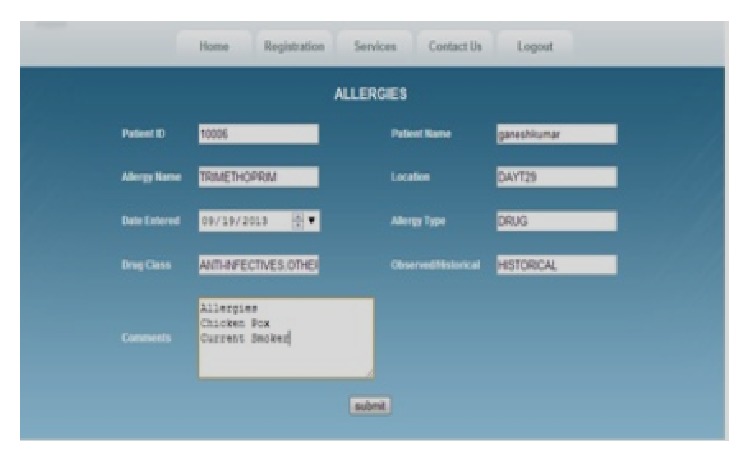
Attributes in the allergies table.

Figure 5 shows the various attributes available in the allergies table. The other tables have different attributes.

### 3.2. Access Control

**Figure 6:**
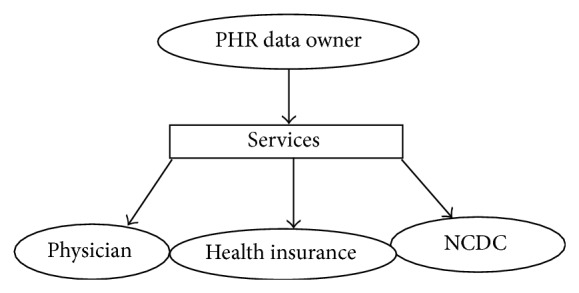
Access control.

The PHR data owner has to give permission to access the data to all potential users based on their requirements. Such users include various healthcare professionals as well as a number of medical insurance companies or organizations. Here, we describe providing attribute-based access control. Initially, the system requires that some of the user's attributes are obtained before the PHR data can be accessed. The system provides access rights to that user based on those attributes. If any one of the user's attributes fails to match predefined values, the user will be denied access to the data. At the same time, the system gives permission such that authorized users can access only certain parts of the PHR data: they cannot access other data areas in the database. Having accessed the PHR data, authorized users obtain a final report, which contains original data for the attributes to which they have access permission. Authorized users obtain only perturbed data for all other attributes. In our application, we allow three types of user access to the PHR data based on their permission level, as shown in Figure 6: the users are physicians, insurance company representatives, and national centre for disease control (NCDC). We have conducted an experiment based on the above setup using a local server and the Amazon EC2 virtual server.

**Figure 7:**
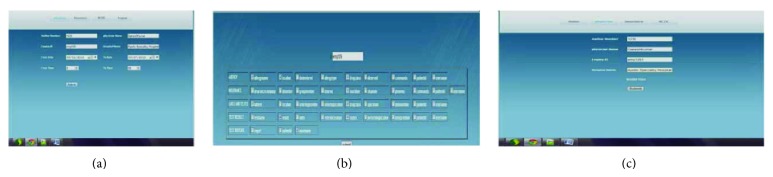
(a) Attributes of a physician with the employee ID number emp126. (b) Permission given to physician emp126. (c) Attributes of physician emp126 for accessing PHR data.

**Figure 8:**
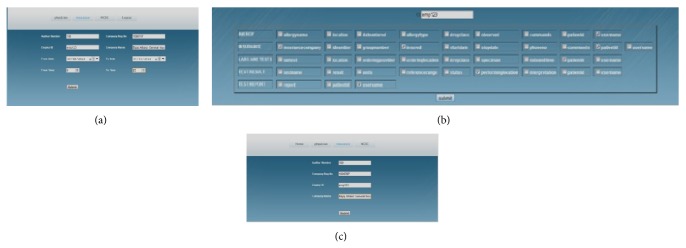
(a) Attributes of insurance company employee with the ID number emp123. (b) Permission granted to insurance employee emp123. (c) Attributes of insurance employee emp123 for accessing PHR data.

Figures 7 and 8 show the attribute-based access control given to physicians and insurance company representatives, respectively, in our system.

Figure 7(a) shows the defining various attributes for a physician with the employee ID number emp126 accessing data in the PHR database. This step was performed by the PHR data owner.

Figure 7(b) shows that permission has been given to various attributes for all five original tables for physician emp126. This access control has been assigned by the PHR data owner.

Figure 7(c) shows that before accessing the data in the PHR database the physician has to provide various values. The physician has already received permission to access the data, and now the physician is entering details in all the required fields. However, because the physician gave the employee ID as emp1261 instead of emp126, the system does not recognize that individual as a valid user. Therefore, the physician is unable to access the data.

Figure 8(a) shows various defining attributes for an insurance company employee with the ID number emp123 in accessing data in the PHR database. This step has been performed by the PHR data owner.

Figure 8(b) shows permission being granted to various attributes for all five original tables to the insurance company employee emp123. This access control has been assigned by the PHR data owner.

Figure 8(c) shows that before accessing the data in the PHR database the insurance company employee has to provide all these values. The employee has already received permission to access the data because all the required fields have been entered correctly.

### 3.3. GDP Algorithm

GDP can be redefined in (1) as follows:

(2) $$\begin{array}{c} \text{GDP}\;\text{Data}=XR+T+\text{G}.\text{N}. \end{array}$$

In (2), *X* signifies the original data, *R* the random rotation matrix, *T* the transpose matrix, and G.N random Gaussian noise. In our system, we have defined five tables for original PHR data. We can select any one of those tables for our process. All values available in the chosen table can be represented in the matrix format defined as *X*. The GDP algorithm can be applied only to numerical data: we convert all original data to numerical data by calculating the equivalent ASCII values. String data conversion involves totaling the ASCII values in that string. After conversion, we have the equivalent numerical value for all original data available in matrix *X*. We then generate the random matrix (*R*) as the size of the original data *X*. In our system, all the values in the random matrix should be from 1.0 to 9.0. We rotate the random matrix vertically clockwise. We then calculate the product of the rotation matrix (*R*) and original data matrix (*X*). Next, we transpose the random matrix, which means shifting the matrix rows from bottom to top. We generate random Gaussian noise as the size of the original data *X*. We take all values in the Gaussian noise matrix from 0.0 to 1.0. Finally, we add the product of rotation matrix (*R*), original data (*X*), transpose matrix (*T*), and Gaussian noise (G.N). We compute the geometrically perturbed data from the original data. These geometrically perturbed data can then be outsourced to the cloud server.

**Figure 9:**
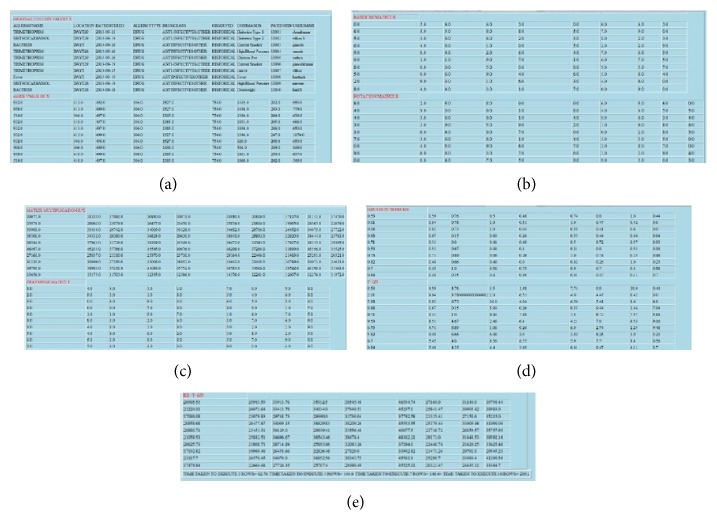
(a) Original PHR data and equivalent numerical value. (b) Random matrix and rotation matrix. (c) Matrix multiplication *R*∗*X* and transpose matrix *T*. (d) Gaussian noise (G.N) and *T* + G.N value. (e) Geometrically perturbed data.

Figure 9(a) represents the original data value used in our application and equivalent numerical value. Figures 9(b), 9(c), 9(d), and 9(e) represent the various computations involved in GDP algorithm.

Figure 9(e) represents the geometrically perturbed data in our system. These data are outsourced to the cloud server. It is difficult to find the original data from these geometrically perturbed data.

### 3.4. Data Retrieval and Query Processing

**Figure 10:**
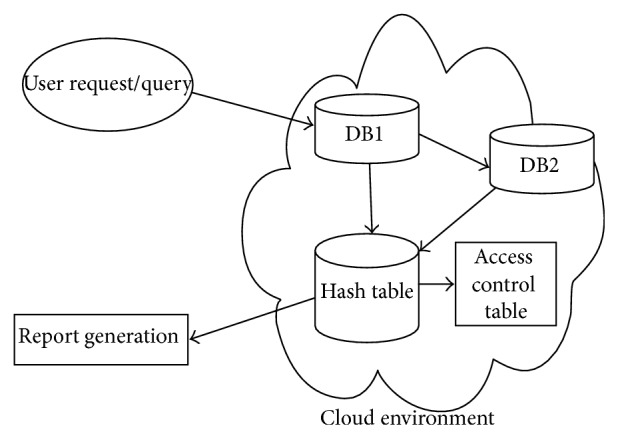
Data retrieval and query processing architecture.

Data retrieval is one of the challenging tasks in the information technology field. Many data retrieval techniques have been discussed [30]. Data retrieval and the query processing architecture in our system appear in Figure 10. The system consists of two databases—DB1 and DB2. DB1 contains only two columns, which are geometrically perturbed values and original data. DB2 is the perturbed database, which is outsourced to the cloud server by the PHR data owner. DB2 consists of perturbed values for all the attributes available in the original data table. A hash table is constructed from DB1 using a hash map. Initially, the user submits a query to the system. This query value checks DB1 and takes the perturbed value from DB1. This perturbed value checks the DB2 and retrieves all the attributes in that row: all those attributes are perturbed values only. Those perturbed values go to the hash table. Based on the concept of the hash map, the original data can be extracted from all the perturbed values. Before generating the report for the user, the system checks the access control table as to whether the user has access to the attributes in their questions. Users can obtain original data just for the attributes to which they have access; otherwise, only perturbed values appear in the report. Our current research efforts are directed at finding an algorithm for efficient data retrieval from the perturbed values:

 DB1, database with two columns (perturbation values and original data);
 DB2, perturbed database (outsourced to the cloud from the local server after GDP).

## 4. Implementation Results

**Figure 11:**
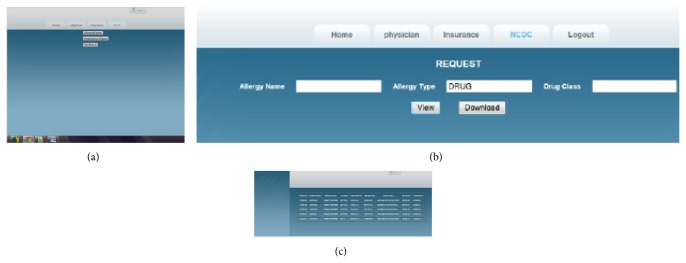
(a) Report selection for physician emp126. (b) Query submission for physician emp126. (c) Allergy report for physician emp126.

**Figure 12:**
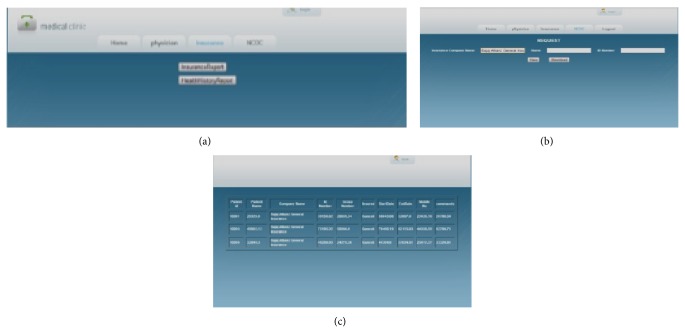
(a) Report selection for insurance company employee emp123. (b) Query submission for insurance company employee emp123. (c) Insurance report for insurance company employee emp123.

We implemented our work on a local server and on the Amazon EC2 virtual server. Figures 11 and 12 show our implementation results and the report generation for physician and insurance company users.

Figure 11(a) presents the report selection option given to the previously authorized physician—emp126. The physician now has three choices in his reports: allergy report, medical history report, and test results. He can choose any one of the reports at a time. After selecting one of the reports, he has to submit a query for the required report generation.

Figure 11(b) shows the query submission screen for the allergy report for physician emp126. In this figure, there are three query fields: allergy name, allergy type, and drug class. The physician may submit any one field or he may submit all three fields. Based upon the query submitted, the report can be generated.

Figure 11(c) shows the allergy report for the query submitted by physician emp126. In this report, the physician obtains the original values for the attributes relating to the patient's ID, allergy name, and drug class. The PHR data owner has already given permission to emp126 to access these fields, as seen in Figure 7(b). For all the other fields, the physician will obtain perturbed data.

Figure 12(a) shows the report selection option given to the previously authorized insurance company employee with ID number 126. The employee now has two choices in the reports: insurance report and medical history report. He can choose any one of the reports at a time. After selecting one of the reports, the employee has to submit a query for their required report generation.

Figure 12(b) shows the query submission screen for the insurance report by company employee emp123. In this figure, there are three fields: insurance company name, name of the person making the query, and their ID number. The employee may submit any one field or all three fields in the query submission screen. Based upon the query submitted, the report can be generated.

Figure 12(c) shows the insurance report for the query submitted by insurance company employee emp123. In this report, the company obtains the original values for the attributes pertaining to the patient's ID, the name of the insurance company, and the employee's name. The PHR data owner has already given permission to the insurance company employee for access to the indicated in Figure 8(b). For all other fields, the insurance company employee will obtain perturbed data.

In our system, NCDC is the authorized user. NCDC obtains access control permission from the PHR data owner. However, the PHR data owner did not give permission to access the original data for any of the attributes. NCDC may access the report, where all the values appear as perturbed data.

**Figure 13:**
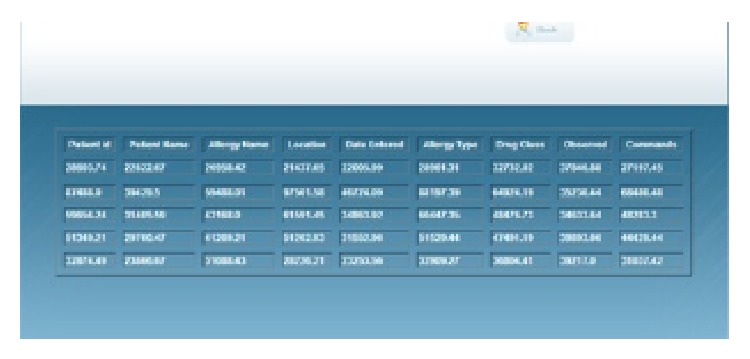
Allergy report for NCDC.

Figure 13 shows the allergy report for the query submitted by NCDC. All the attributes are perturbed values.

## 5. Performance Analysis

In this section, we analyze the performance of our proposed system. Here we calculate time taken for data perturbation. We calculate perturbation time for different size of PHR records. By changing the number of records, we measure the time taken for perturbation. This calculated perturbation time compared with AES encryption time for our system.  Table 1 shows time taken to encrypt PHR data using AES and time taken to perturb PHR data using GDP.

**Figure 14:**
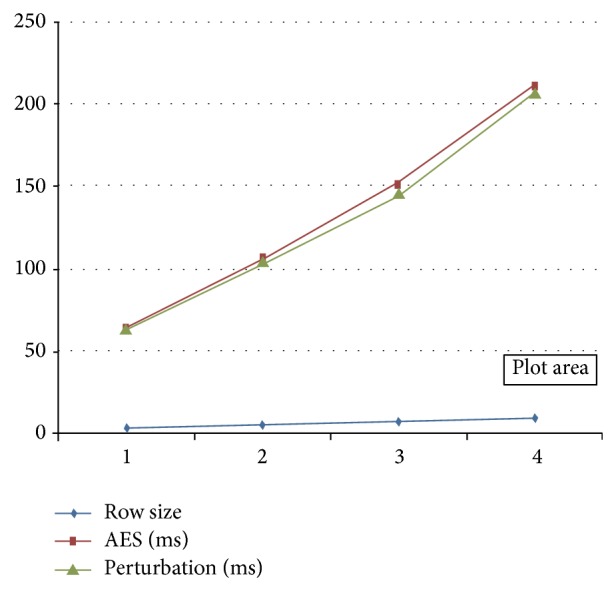
AES encryption time and GDP perturbation time.

Figure 14 shows the comparison between encryption time using AES and perturbation time using GDP. From this figure, we find there is small decrease in the time by using GDP compared with AES. By changing the record size, encryption time and perturbation time also increase. In *x*-axis, we take the record size value as 3, 5, 7, and 10. In *y*-axis, we take the encryption time using AES and perturbation time using GDP.

## 6. Conclusion

Cloud computing is becoming a part of everyday life. Cloud technology is being proposed for different applications, such as in the areas of consumer electronics, finance, healthcare, and business. Recent surveys have found that major resistance factors for organizations moving to the cloud are data privacy and security concerns. Security and the privacy of outsourced data are indeed major challenges in cloud computing. Most approaches towards solving the problem of data privacy and security are based on cryptographic encryption and decryption techniques. In this study, we propose the first GDP-based PHR data transactions in cloud computing. We have implemented and demonstrated a system that preserves privacy of PHR data transactions using GDP. We allow a PHR data owner to securely move PHR data to cloud servers, and the performance of the system is guaranteed even in the case of large databases and large numbers of user queries. We have reduced the complexity of key management in using cryptographic techniques. We used GDP to perturb PHR data before outsourcing to the cloud server. In future studies, we aim to implement medical image and multimedia data transactions using GDP in cloud computing and improve the performance of data retrieval and query processing.

## Figures and Tables

**Table 1:** Comparison between AES encryption time and GDP perturbation time.

Serial number	Record size	Time taken to encrypt PHR data using AES (ms)	Time taken to perturb PHR data using GDP (ms)
1	3	63.54	62.76
2	5	105.24	104.6
3	7	152.45	146.44
4	10	212.78	209.2
